# Gastrointestinal Helminth Infections in Captive Wild Animals of Bangladesh: Implications for Zoo Health Management and Zoonotic Risk

**DOI:** 10.1155/japr/1056182

**Published:** 2026-05-30

**Authors:** Md. Shahriar Rahman, Jarin Tasnim, Md. Shakil Mahmud Supto, Tarek Siddiki, H. M. Shahadat, Tilak Chandra Nath

**Affiliations:** ^1^ Department of Parasitology, Sylhet Agricultural University, Sylhet, Bangladesh, sau.ac.bd; ^2^ Faculty of Veterinary, Animal and Biomedical Sciences, Sylhet Agricultural University, Sylhet, Bangladesh, sau.ac.bd; ^3^ Rangpur Zoo, Rangpur, Bangladesh

**Keywords:** Bangladesh, captive wildlife, gastrointestinal helminths, parasitic infections, zoo epidemiology, zoonotic risk

## Abstract

Gastrointestinal helminth (GIH) infections remain a significant but undercharacterized threat to captive wildlife health and may contribute to zoonotic transmission at the human–animal interface, particularly in Bangladesh, where zoological facilities are located near densely populated urban centers. This study investigated the prevalence, diversity, and host distribution of GIHs among captive wild animals in two major facilities—the Bangladesh National Zoo and Tilagarh Eco Park, Sylhet—between May and December 2023. In a cross‐sectional design, 80 fecal samples from mammals, birds, and reptiles were analyzed using a modified formalin–ether sedimentation technique, and parasites were identified based on morphological criteria. Overall, 51.25% (41/80) of animals were infected with at least one helminth species. Five helminth taxa were detected, including members of Ascarididae and Capillarinae with known zoonotic relevance. Infection prevalence varied by host group, with the highest burden observed in reptiles (100%), followed by herbivorous mammals, whereas carnivorous and omnivorous mammals had lower prevalence (16.67%). Mixed infections occurred in 27.78% of infected animals. The low occurrence of trematodes and cestodes likely reflects the limited availability of intermediate hosts under captive conditions. Despite the absence of overt clinical signs, the high prevalence suggests substantial subclinical infection that may compromise animal welfare and increase zoonotic risk. These findings highlight critical gaps in parasitological surveillance and management in captive wildlife systems and underscore the need for integrated One Health approaches, including routine monitoring, improved husbandry, and targeted parasite control strategies to mitigate infection risks at the human–animal interface.

## 1. Introduction

Bangladesh is characterized by rich biodiversity and diverse ecosystems that support a wide range of wildlife species. In response to global biodiversity loss and habitat degradation, wildlife conservation efforts have increasingly relied on the establishment of protected areas, zoological gardens, and wildlife parks to conserve threatened species, facilitate captive breeding, and promote public awareness of conservation issues [1, 2]. Zoological institutions, in particular, serve as important reservoirs for ex situ conservation, scientific research, education, and, in some cases, species reintroduction programs [3]. However, the confinement of wild animals under artificial conditions presents unique health challenges, among which parasitic diseases remain a major concern.

Gastrointestinal parasitism is one of the most frequently reported health problems affecting captive wild animals worldwide [4, 5]. Helminth infections can impair growth, reproduction, and overall fitness, and in severe cases may result in morbidity or mortality, thereby undermining conservation and animal welfare goals [6, 7]. The risk of parasitic infection is often amplified in captive settings due to factors such as high stocking density, limited enclosure space, close proximity of multiple species, repeated exposure to contaminated environments, and inadequate sanitation [8]. Additionally, chronic stress associated with captivity has been shown to suppress immune responses, rendering animals more susceptible to parasitic infections and facilitating persistent or subclinical disease states [9].

Beyond animal health implications, gastrointestinal parasites of captive wildlife are increasingly recognized for their zoonotic significance. Wildlife has historically played a pivotal role in the emergence and transmission of infectious diseases to humans, particularly at interfaces where humans, domestic animals, and wildlife interact closely [10]. Globally, more than two‐thirds of wildlife populations declined between 1970 and 2016, largely due to habitat loss, urban expansion, and intensified human–animal interactions, all of which increase opportunities for pathogen spillover [11]. Several studies have documented the presence of zoonotic gastrointestinal parasites in captive wild animals, posing potential health risks to zoo personnel, visitors, and surrounding communities [12–14].

In Bangladesh, most zoological gardens and wildlife parks are situated near densely populated urban areas for logistical and economic reasons, resulting in frequent and close contact between humans and captive animals [13]. Limited physical barriers, high housing density, and shared environmental exposure further heighten the risk of parasite transmission within and beyond zoo boundaries. In this context, captive wild animals may act as sentinels for emerging infectious diseases that threaten humans, domestic animals, and free‐ranging wildlife [15]. Addressing such risks requires an integrated One Health approach that recognizes the interconnectedness of animal health, human health, and ecosystem integrity [3].

Despite these concerns, comprehensive epidemiological data on gastrointestinal helminth (GIH) infections in captive wild animals in Bangladesh remain scarce. Existing studies are limited in scope, and baseline information on prevalence, host distribution, and parasite diversity is insufficient to inform effective control and prevention strategies. To address this knowledge gap, the present study aimed to investigate the occurrence and distribution of GIHs among captive wild animals housed at two zoological facilities in Bangladesh. The findings are expected to provide baseline epidemiological evidence to support targeted parasite control programs, improve zoo health management, and contribute to the mitigation of zoonotic risks within a One Health framework.

## 2. Materials and Methods

### 2.1. Study Area and Study Design

**Figure 1:**
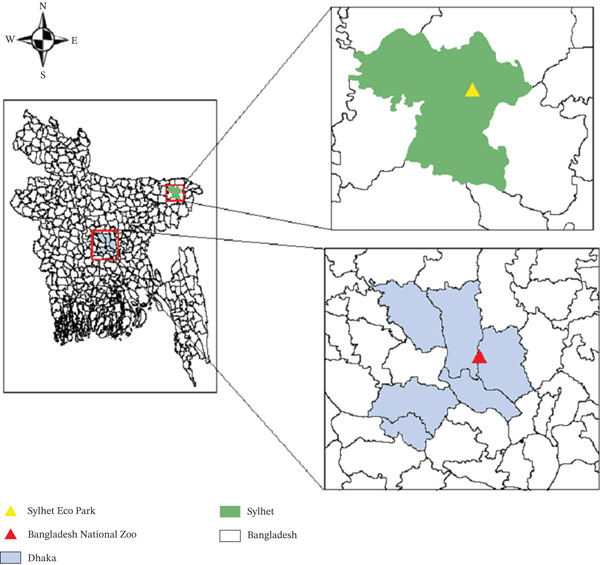
Study area.

A cross‐sectional parasitological study was conducted between May and December 2023 at two major captive wildlife facilities in Bangladesh: the Bangladesh National Zoo, located in Dhaka, and Tilagarh Eco Park, situated in Sylhet. These institutions house a diverse assemblage of captive wild animals under varying management and enclosure conditions and are located in proximity to densely populated urban areas. The geographical locations of the study sites are presented in Figure 1. Laboratory examinations were carried out at the Parasitology Laboratory, Sylhet Agricultural University, Bangladesh.

### 2.2. Study Population and Sampling Strategy

The study population comprised captive wild animals representing mammals, birds, and reptiles maintained at the selected zoological facilities. A total of 80 fresh fecal samples were collected from individual animals belonging to different taxonomic groups. The sampled species included Royal Bengal tiger (*Panthera tigris tigris*, *n* = 5), black bear (*Ursus thibetanus*, *n* = 3), lion (*Panthera leo*, *n* = 4), striped hyena (*Hyaena hyaena*, *n* = 3), jackal (*Canis aureus*, *n* = 3), Asian elephant (*Elephas maximus*, *n* = 5), spotted deer (*Axis axis*, *n* = 10), zebra (*Equus quagga*, *n* = 7), rhesus macaque (*Macaca mulatta*, *n* = 13), Indian crested porcupine (*Hystrix indica*, *n* = 1), peacock (*Pavo cristatus*, *n* = 5), macaw (*Ara ararauna*, *n* = 3), African grey parrot (*Psittacus erithacus*, *n* = 4), silver pheasant (*Lophura nycthemera*, *n* = 3), western swamphen (*Porphyrio porphyrio*, *n* = 3), Bengal vulture (*Gyps bengalensis*, *n* = 3), pigeon (*Columba livia*, *n* = 3), and python (*Python molurus*, *n* = 2). Animals were categorized according to taxonomic class and feeding habits to facilitate comparative analysis of helminth prevalence across host groups.

### 2.3. Fecal Sample Collection and Preservation

Fresh fecal samples were collected early in the morning from individual enclosures with the assistance of trained animal caretakers to ensure accurate attribution to each animal and to minimize environmental contamination. Samples were collected immediately after defecation using clean disposable tools and transferred into labeled, leak‐proof stool containers containing 10% formalin as a preservative. Each container was clearly labeled with species identification, date, and sampling location. The samples were placed in sealed biohazard bags and transported under appropriate conditions to the laboratory for parasitological examination.

### 2.4. Parasitological Examination

Upon arrival at the laboratory, each sample was assigned a unique identification number and divided into two aliquots, one for immediate parasitological analysis and the other reserved for quality control and cross‐verification. All specimens were processed using modified formalin–ether concentration test (FET) and sodium nitrate flotation (SNF) method following established protocols. For FET, approximately 0.5 g of feces was mixed with 10 mL of normal saline in a glass container and stirred thoroughly. The mixture was strained through two layers of gauze into a 15‐mL centrifuge tube. Next, 2.5 mL of 10% formaldehyde and 1 mL of ether were added. The solution was mixed well and centrifuged at 1000 rpm for 3 min. The supernatant was discarded, and slides were prepared from the sediment. Two slides were prepared (one with saline and the other with iodine), covered with a cover slip, and examined under a microscope [16, 17]. For SNF, approximately 1 g of stool was mixed with 10 mL of saturated sodium nitrate solution in a centrifuge tube. The mixture was strained through a sieve to remove debris and then centrifuged at 1500 rpm for 5 min. After centrifugation, the tube was filled with sodium nitrate solution until a meniscus formed at the top. A coverslip was placed on the tube and left for 10 min to allow eggs to float to the surface. The coverslip was then transferred to a slide and examined under a microscope [18]. In cases where *Strongyloides* spp. or hookworm infection was suspected, agar plate culture was employed to enhance larval recovery, with samples incubated on nutrient agar at 27°C–35°C for 4–5 days, enabling the detection of filariform larvae and free‐living adult stages. Helminth species identification was performed based on comprehensive morphological assessment, including egg size, shape, shell structure, embryonation status, and larval characteristics, using multiple complementary and widely recognized taxonomic keys and diagnostic references. These included Sepulveda and Kinsella [19], Garcia [16], WHO [18] guidelines, and additional contemporary parasitological literature alongside Soulsby [20], thereby strengthening methodological rigor, enhancing diagnostic accuracy, and improving the reproducibility and reliability of species identification.

### 2.5. Data Collection on Management Practices

To complement parasitological findings, data on animal management and parasite control practices were collected using a structured questionnaire administered to animal caretakers and veterinary personnel. A total of 10 animal caretakers and one veterinary officer participated in the survey. The questionnaire was designed to assess knowledge, attitudes, and practices related to parasite control, enclosure hygiene, and animal health management. Prior to data collection, the objectives of the study were explained to all participants, and informed verbal consent was obtained. When necessary, questions were translated or explained in the local language to ensure accurate understanding and reliable responses. Additional information was obtained from official zoo record books to verify deworming history and management practices.

### 2.6. Data Analysis

Data obtained from laboratory examinations and questionnaires were entered into Microsoft Excel and analyzed using descriptive statistical methods. Prevalence was calculated as the proportion of animals infected with one or more GIHs relative to the total number examined. Results were summarized according to host species, taxonomic group, and feeding habit.

## 3. Results

### 3.1. Overall Prevalence of GIH Infections

**Figure 2:**
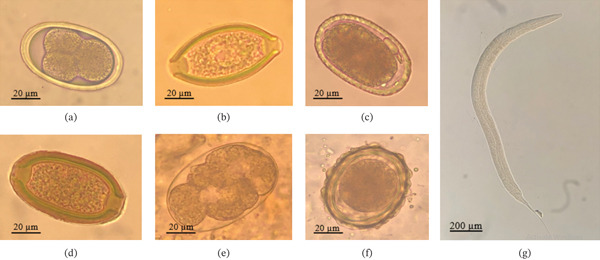
Identified endoparasites in captive wildlife: (a) Ascarididae (Bengal Vulture); (b) *Trichuris* sp. (Peacock); (c) Ascarididae (Silver Pheasant); (d) Capillarinae (Deer); (e) Hookworm (Jackal); (f) Ascarididae (Zebra); (g) *Strongyloides* larvae (Monkey).

A total of 80 fecal samples collected from captive wild animals were examined for GIHs. Of these, 41 samples were positive, yielding an overall prevalence of 51.25% (Table 1). GIH infections were detected in all major host groups included in the study, namely mammals, birds, and reptiles, although the prevalence varied substantially among these groups. Helminths detected in birds included Ascarididae, Capillarinae, *Trichuris* spp., and *Strongyloides* spp. (Table 2; Figure 2). Reptiles exhibited the highest prevalence, with 100% (2/2) of the examined samples testing positive for helminth infection. Birds showed a high prevalence of 62.50% (15/24), whereas mammals demonstrated a comparatively lower prevalence of 44.44% (24/54). Within mammals, herbivorous species and primates had a markedly higher infection rate (58.33%, 21/36) than carnivorous and omnivorous mammals (16.67%, 3/18).

**Table 1:** Prevalence of gastrointestinal helminths among captive wild animals by host group.

Host group	No. examined	No. positive	Prevalence (%)
Mammals	54	24	44.45
Birds	24	15	62.50
Reptiles	2	2	100.00
Overall	80	41	51.25

**Table 2:** Distribution of gastrointestinal helminths taxa detected in captive wild animals.

Helminth taxon	Mammals	Birds	Reptiles	Total positive hosts (*n*)
Ascarididae	✓	✓	✓	High
Capillarinae	✓	✓	—	Moderate
*Strongyloides* spp.	✓	✓	—	Low
*Trichuris* spp.	—	✓	—	Low
Hookworms	✓	—	—	Low

*Note:* ✓ = detected; – = not detected.

### 3.2. Distribution of Helminth Infections Among Mammalian Host Groups

GIH infections were detected across several mammalian orders (Table 3). Among Artiodactyla, represented by spotted deer (*A. axis*), 60.00% (6/10) of animals were positive for helminths. Perissodactyla, represented by zebras, had a prevalence of 71.42% (5/7), whereas Proboscidea, represented by Asian elephants, showed a prevalence of 60.00% (3/5). Among primates, 53.84% (7/13) of rhesus macaques were positive for GIHs. No helminth infections were detected in black bears, lions, Royal Bengal tigers, striped hyenas, or Indian crested porcupines. Among carnivorous mammals, infections were observed only in jackals, with a prevalence of 100% (3/3). Hookworms were the only helminths identified in carnivorous mammals, whereas herbivorous mammals and primates harbored Ascarididae eggs, Capillarinae eggs, and *Strongyloides* spp.

**Table 3:** Species‐wise prevalence of gastrointestinal helminths in mammals.

Mammalian order	Species examined	No. examined	No. positive	Prevalence (%)	Predominant helminths
Carnivora	Tiger, lion, bear, hyena, and jackal	18	3	16.67	Hookworms
Artiodactyla	Deer	10	6	60.00	Ascarididae, Capillarinae
Perissodactyla	Zebra	7	5	71.42	Ascarididae, Capillarinae
Proboscidea	Elephant	5	3	60.00	Ascarididae
Primates	*Rhesus macaque*	13	7	53.84	*Strongyloides* spp.

### 3.3. Occurrence of GIHs in Avian Species

Among avian hosts, 62.50% (15/24) of the examined samples were positive for GIHs. Infection prevalence varied by species (Table 4). All examined peafowl, macaws, and silver pheasants were infected, yielding a prevalence of 100% in these species. Moderate prevalence was observed in Bengal vultures at 66.67% (2/3), whereas gray parrots and pigeons exhibited lower prevalence rates of 25.00% (1/4) and 33.33% (1/3), respectively. No helminth infection was detected in western swamphens.

**Table 4:** Species‐wise prevalence of gastrointestinal helminths in birds.

Avian species	No. examined	No. positive	Prevalence (%)	Helminths detected
Peacock (*Pavo cristatus*)	5	5	100	Ascarididae, Capillarinae, *Trichuris*
Macaw (*Ara ararauna*)	3	3	100	Capillarinae, *Strongyloides*
Silver pheasant (*Lophura nycthemera*)	3	3	100	Ascarididae, Capillarinae
Gray parrot (*Psittacus erithacus*)	4	1	25	Capillarinae
Bengal vulture (*Gyps bengalensis*)	3	2	66.67	Ascarididae
Pigeon (*Columba livia*)	3	1	33.33	Capillarinae
Western swamphen (*Porphyrio porphyrio*)	3	0	0	—

### 3.4. GIH Infections in Reptiles

Reptiles were represented exclusively by pythons, all of which were positive for GIHs, resulting in a prevalence of 100% (2/2) (Table 1). The only helminth taxon detected in reptiles was Ascarididae (Table 2).

### 3.5. Frequency and Patterns of Single and Mixed Helminth Infections

Among the 41 helminth‐positive animals, single‐species infections predominated, accounting for 72.22% (30/41) of cases, whereas mixed helminth infections were detected in 27.78% (11/41) of animals (Table 5). Mixed infections were more common in birds (46.67%, 7/15) than in mammals (25.00%, 6/24) and were not observed in reptiles. In mammals, mixed infections primarily involved combinations of Ascarididae eggs and *Capillaria* spp., particularly in deer and zebras. In birds, mixed infections included combinations of Ascarididae, Capillarinae, *Trichuris*, and *Strongyloides*, with the highest diversity of mixed infections observed in peafowl and silver pheasants (Table 5).

**Table 5:** Frequency of single and mixed gastrointestinal helminth infections.

Host group	No. positive	Single infection *n* (%)	Mixed infection *n* (%)
Mammals	24	18 (75.00)	6 (25.00)
Birds	15	8 (53.33)	7 (46.67)
Reptiles	2	2 (100.00)	0 (0.00)
Total	41	30 (72.22)	11 (27.78)

## 4. Discussion

GIH infections represent a persistent challenge to the health management of captive wild animals and may undermine both animal welfare and conservation objectives in zoological institutions. In the present study, more than half of the examined animals (51.25%) were infected with at least one GIH species, highlighting a substantial infection burden within the studied facilities. This prevalence is comparable to reports from other zoological parks in Asia and Africa, where helminth infections have been documented as common despite routine veterinary care [1, 21, 22].

The marked variation in prevalence among host groups observed in this study underscores the influence of host ecology, feeding behavior, and enclosure management on parasite transmission. Reptiles exhibited the highest prevalence (100%), although this finding should be interpreted cautiously due to the small sample size. Similarly high prevalence rates in captive reptiles have been reported elsewhere and are often attributed to persistent environmental contamination and limited enclosure sanitation [12]. Birds also demonstrated a high prevalence (62.50%), with several species showing universal infection, whereas mammals exhibited a comparatively lower overall prevalence. Within mammals, herbivores and primates were significantly more affected than carnivorous and omnivorous species, a pattern that has been consistently reported in zoo‐based parasitological studies [4, 5].

The high infection rates observed among herbivorous mammals such as deer, zebras, and elephants likely reflect their frequent contact with contaminated substrates, grazing behavior, and prolonged exposure to infective stages in soil and enclosure environments. In contrast, the low prevalence among carnivorous mammals, with infections confined exclusively to jackals, may be related to differences in feeding regimes, enclosure design, and husbandry practices. The detection of hookworms solely in jackals aligns with previous findings that identify carnivores as important reservoirs for soil‐transmitted helminths with zoonotic potential [23, 24].

The diversity of helminths detected, including Ascarididae, Capillarinae, *Strongyloides* spp., *Trichuris* spp., and hookworms, further emphasizes the complexity of parasite transmission dynamics in captive environments. Most of these parasites are transmitted via the fecal–oral route, and their presence indicates ongoing environmental contamination within enclosures. Similar parasite assemblages have been reported in captive wildlife elsewhere, reinforcing the role of contaminated food, water, soil, and fomites in sustaining transmission cycles [12, 21]. Zoo personnel and visitors may inadvertently act as mechanical vectors through footwear, clothing, equipment, or direct contact, thereby facilitating parasite dissemination [25].

Mixed helminth infections were detected in more than one‐quarter of infected animals, with a notably higher frequency in birds compared to mammals. This finding is consistent with previous reports suggesting that zoo birds are particularly susceptible to mixed infections due to communal housing, shared feeding areas, and the broad host specificity of many avian nematodes [25]. The frequent detection of Ascarididae and Capillarinae in birds is noteworthy, as members of these groups are associated with gastrointestinal disturbances such as intestinal obstruction, weight loss, and reduced fitness in avian hosts [26, 27]. However, identification was limited to the family or subfamily level based on fecal egg morphology, and no species‐level attribution was made. In particular, species such as *Capillaria hepatica* were not considered, as their eggs are not typically shed in feces but are released into the environment following host decomposition [28]. Therefore, the findings should be interpreted within the constraints of the diagnostic methodology employed.

The absence or low detection of trematode and cestode infections in this study is likely attributable to the indirect life cycles of these parasites, which require one or more intermediate hosts that are typically scarce or absent in captive settings [29, 30]. However, incomplete physical barriers between zoo enclosures and surrounding environments, as observed during site assessments, may still permit the introduction of intermediate hosts and should not be overlooked as a potential risk factor.

The persistence of helminth infections despite reported routine anthelmintic administration raises concerns regarding the effectiveness of current parasite control strategies. Inadequate dosing, improper drug rotation, lack of targeted treatment, and rapid reinfection from contaminated environments may all contribute to sustained transmission. Moreover, repeated use of broad‐spectrum anthelmintics without parasitological monitoring may promote the development of drug resistance, as previously documented in captive wild ruminants [31]. These findings highlight the necessity of revising parasite management programs to incorporate regular fecal surveillance, evidence‐based anthelmintic use, and improved sanitation practices.

**Figure 3:**
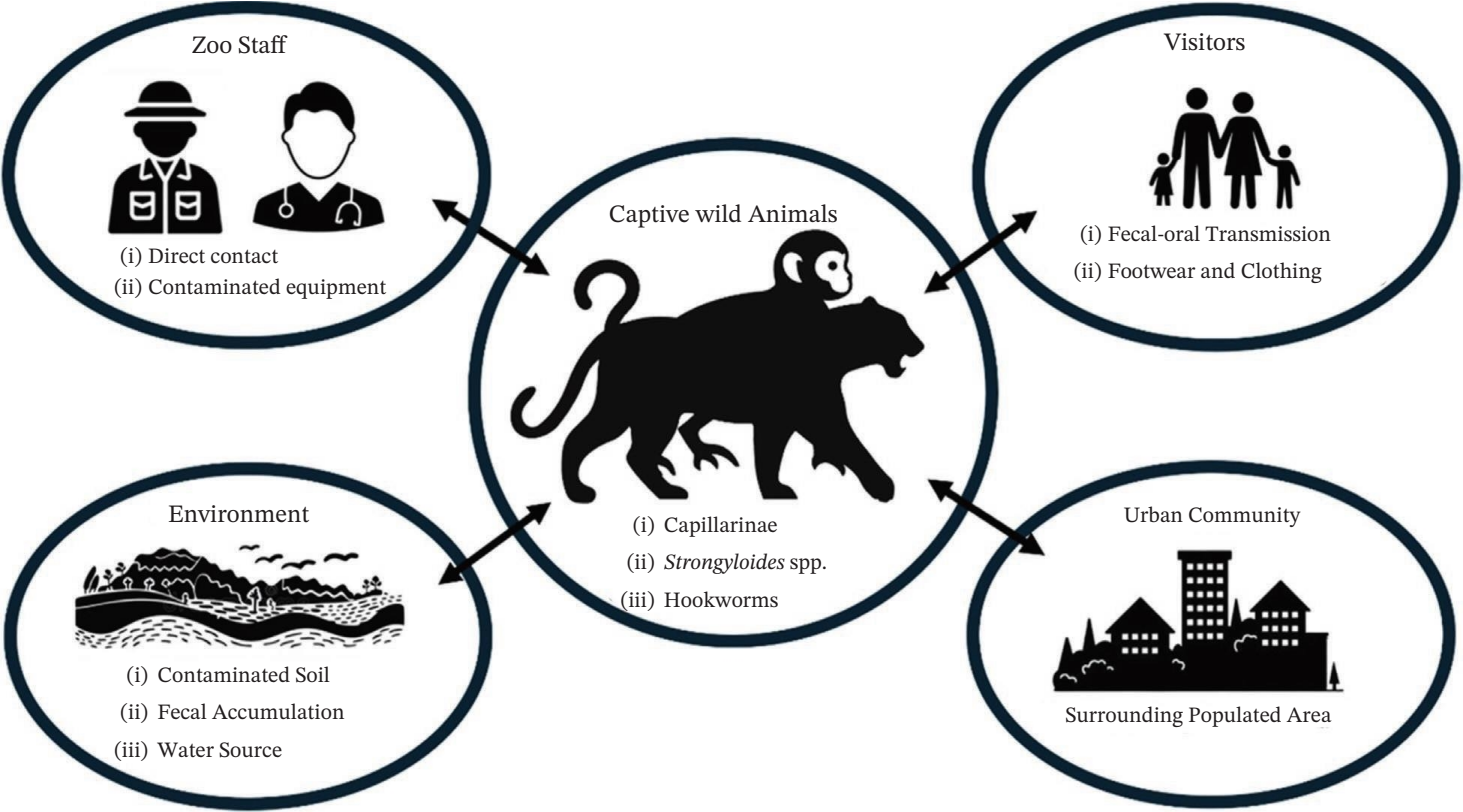
Zoonotic risk of gastrointestinal helminths in captive wildlife.

From a One Health perspective, the detection of helminths such as *Capillaria* spp., *Strongyloides* spp., and hookworms should be interpreted with caution, as these genera include both zoonotic and nonzoonotic species, and species‐level identification was not established in this study. Therefore, no direct inference regarding zoonotic risk can be made based solely on the present findings (Figure 3). Nonetheless, maintaining standard hygiene practices, appropriate use of personal protective equipment, and routine health monitoring remain important in zoological settings to support overall animal and human health.

Several limitations should be considered when interpreting the results of this study. The relatively small sample size and restricted number of zoological facilities may limit the generalizability of the findings to all captive wildlife populations in Bangladesh. The cross‐sectional design provides only a snapshot of infection status and does not capture seasonal variations or longitudinal trends. Additionally, parasite identification relied on morphological methods, which may lack the resolution of molecular techniques for species‐level confirmation. Future studies incorporating molecular diagnostics, larger sample sizes, and longitudinal monitoring would provide deeper insights into parasite ecology and transmission dynamics in captive wildlife.

Despite these limitations, the present study provides valuable baseline epidemiological data on GIH infections in captive wild animals in Bangladesh. The findings underscore the need for integrated parasite control strategies that combine routine surveillance, improved husbandry, targeted treatment, and One Health–oriented risk mitigation to protect animal health, public health, and biodiversity conservation.

## 5. Conclusion

This study provides baseline epidemiological evidence that GIH infections are widespread among captive wild animals in Bangladesh, with notable variation across host groups and frequent subclinical and mixed infections. The detection of multiple helminth taxa, including species with zoonotic potential, highlights captive wildlife facilities as important interfaces for parasite persistence and potential spillover. These findings indicate that current parasite control measures may be insufficient and underscore the need for routine parasitological surveillance, improved sanitation, and targeted anthelmintic strategies. From a One Health perspective, strengthening integrated parasite management is essential to protect animal welfare, reduce zoonotic risk, and support sustainable zoo management.

## Author Contributions

All authors reviewed and provided feedback for this manuscript.

## Funding

No funding was received for this manuscript.

## Disclosure

The final version of this manuscript was vetted and approved by all authors.

## Ethics Statement

This study protocol was reviewed and approved by the Sylhet Agricultural University Research System (SAURES‐UGC‐2023‐2024‐02) and the Department of Parasitology, Sylhet Agricultural University, Bangladesh.

## Conflicts of Interest

The authors declare no conflicts of interest.

## Data Availability

The data that support the findings of this study are available from the corresponding author upon reasonable request.
